# Supplementary material for: Mobility of Single Vacancies and Adatoms in Graphene at Room Temperature
Source: Small. 2025 Jul 7;21(35):2504370. doi: 10.1002/smll.202504370 (PMC12410897; doi:10.1002/smll.202504370)
Supplement: Supplementary file 1 — Supporting Information [file SMLL-21-2504370-s001.docx]

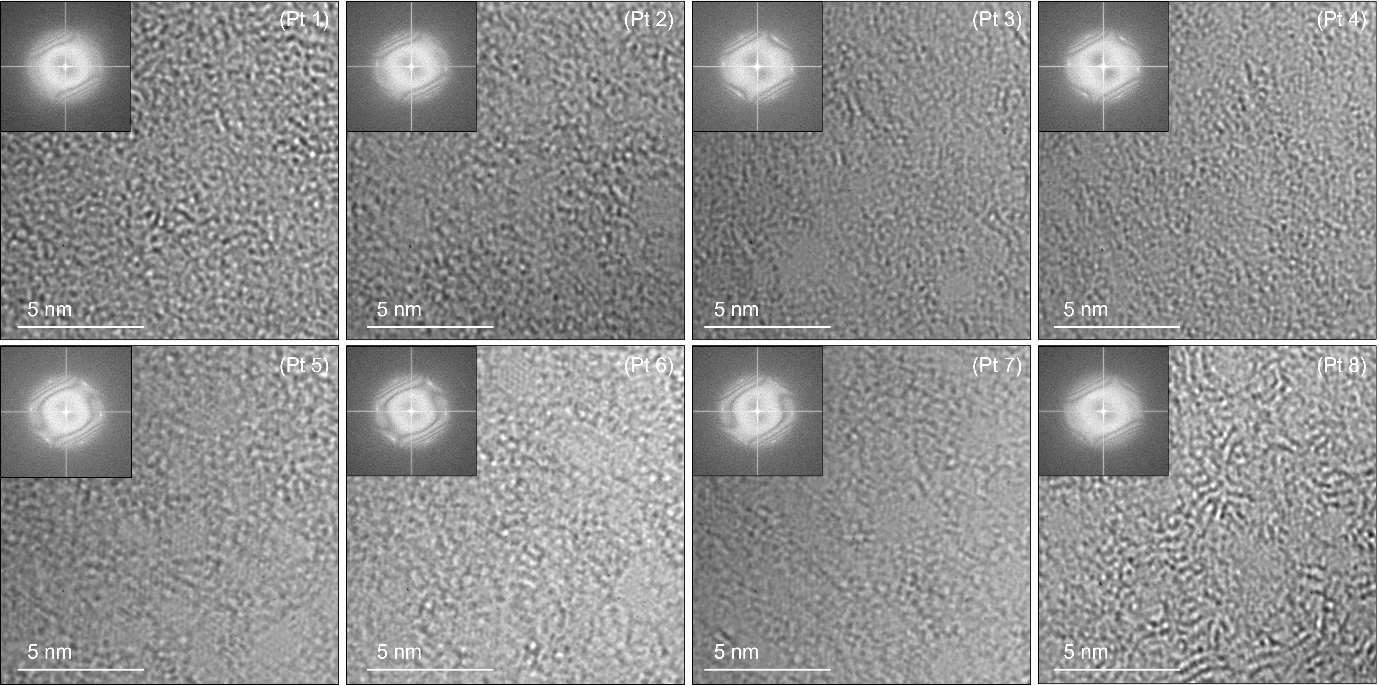


Figure S1: HRTEM images at different position of the graphene lattice marked in Fig. 8a of the main manuscript.
